# Transient Receptor Potential Vanilloid channel regulates fibroblast differentiation and airway remodeling by modulating redox signals through NADPH Oxidase 4

**DOI:** 10.1038/s41598-020-66617-2

**Published:** 2020-06-17

**Authors:** Nosayba Al-Azzam, Lakshminarayan Reddy Teegala, Sabita Pokhrel, Samrawit Ghebreigziabher, Tatiana Chachkovskyy, Sathwika Thodeti, Ignacio Gavilanes, Kayla Covington, Charles K. Thodeti, Sailaja Paruchuri

**Affiliations:** 1 Department of Chemistry, University of Akron, Akron, OH US; 2 Department of Integrative Medical Sciences, Northeast Ohio Medical University, Rootstown, OH US; 3 Department of Physiology and Biochemistry, Jordan University of Science and Technology, Irbid, Jordan

**Keywords:** Cell signalling, Ion channels

## Abstract

Asthma is characterized by pathological airway remodeling resulting from persistent myofibroblast activation. Although transforming growth factor beta 1 (TGFβ1), mechanical signals, and reactive oxygen species (ROS) are implicated in fibroblast differentiation, their integration is still elusive. We identified that Transient Receptor Potential Vanilloid 4 (TRPV4), a mechanosensitive ion channel mediates lung fibroblast (LF) differentiation and *D. farinae*-induced airway remodeling via a novel TRPV4-NADPH Oxidase 4 (NOX4) interaction. NOX4-mediated ROS production is essential for TGFβ1-induced LF differentiation via myocardin-related transcription factor-A (MRTF-A) and plasminogen activator inhibitor 1 (PAI-1). Importantly, TRPV4 inhibition prevented TGFβ1-induced NOX4 expression and ROS production. Both TRPV4 and NOX4 are activated by phosphatidylinositol 3-kinase (PI3K) downstream of TGFβ1, and signals from both TRPV4 and Rac are necessary for NOX4 upregulation. Notably, NOX4 expression is higher in fibroblasts derived from asthmatic patients (disease human LF; DHLF) in comparison to non-asthmatics (normal human LF; NHLF). Further, *NOX4* expression is up-regulated in the lungs of *D.farinae*-treated wild type mice (WT) relative to saline-treated WT, which was attenuated in TRPV4 knockout (KO) mice. Our findings suggest that TRPV4 integrates TGFβ1 and ROS signaling through NOX4 and, TRPV4-NOX4 interaction is amenable to target lung remodeling during asthma.

## Introduction

Asthma is characterized by inflammation, eosinophilic infiltration, bronchial hyperresponsiveness^1,2^, and significant airway remodeling^3,4^. In fact, airway remodeling due to excessive extracellular matrix (ECM) deposition has been shown to be responsible for the rapid decline in lung function experienced in the treatment of resistant asthmatics compared to non-asthmatics^5^. While airway inflammation is known to play a role in the progression of airway remodeling, the mechanisms regulating this process remain poorly understood. In asthmatics, susceptibility to injury and aberrant repair responses result in persistent activation of fibroblasts, differentiating them into myofibroblasts^6^. Myofibroblasts are hyper-secreting, contractile cells that facilitate wound healing through increased ECM synthesis^7–9^. Disproportionate ECM production and prolonged survival of myofibroblasts can lead to pathological fibrosis^10–12^. Soluble factor, Transforming Growth Factor beta 1 (TGFβ1), has been identified as a major player in the differentiation of fibroblasts to myofibroblasts and lung remodeling. However, the downstream signaling pathways and their intersection is still not completely understood.

TGFβ1 promote fibroblast differentiation by enhancing fibrotic and ECM components^13^, leading to abnormal mechanical properties. Mechanical rigidity is also a causal factor in myofibroblast differentiation, compromising lung function^14^. Transient Receptor Potential Vanilloid 4 (TRPV4) is a mechanosensitive ion channel that has been shown to regulate lung function^15^ and airway hyperresponsiveness in patients with asthma^16^, cardiac^17^ and lung^18^ fibrosis. Employing a model of *Dermatophagoides farinae (D.farinae)-*induced asthma, we recently reported that TRPV4 KO mice were protected from *D.farinae -*induced airway remodeling^19^.

Interestingly, in addition to soluble and mechanical signals, enhanced ROS production was well documented in asthma with high levels of superoxide generated by nicotinamide adenine dinucleotide phosphate (NADPH) oxidases (NOX)^20^. Although the NOX family includes seven members (NOX1, NOX2, NOX3, NOX4, NOX5, Duox1 and Duox2), TGFβ1-mediated fibroblast differentiation has been shown to be regulated by NOX4^21^ in LF. Unlike other NOX isoforms, NOX4 has been suggested to be constitutively active and is regulated at the level of expression. Notably, NOX4 expression and activity (ROS production) is demonstrated to be a driving force for fibroblast differentiation^21,22^ and lung fibrosis^21^. However, the mechanism by which NOX4 expression is enhanced in asthmatic airway remodeling remains unclear. Although soluble, mechanical and redox pathways are implicated in fibroblast differentiation and lung remodeling, little is known regarding their integration and contribution to asthma. Here, we demonstrate that NOX4 integrates TGFβ1 and mechanical signaling in fibroblast differentiation and lung remodeling during asthma.

## Materials and Methods

### Animals

C57BL/6J mice (Jackson Laboratories) and TRPV4 KO mice on C57BL/6 background^23^ were maintained at the Comparative Medicine Unit at Northeast Ohio Medical University (NEOMED). All animal experiments were done in accordance with standard guidelines as approved by the Animal Care and Use Committee of NEOMED.

### Reagents

The following chemicals and reagents were purchased commercially: RN1734 (selective TRPV4 inhibitor)^17,24^, EHT 1864 (selective Rac inhibitor)^25^, (Tocris Bioscience, Minneapolis, MN), LY294002 (PI3K inhibitor)^26,27^ (Cayman Chemicals, Ann Arbor, MI), TGFβ1 (R&D Systems, Minneapolis, MN), PAI-1 antibody (Ab) (Cell Signaling Technology, Danvers, MA), Diphenyleneiodonium chloride (DPI; NOX inhibitor), N-Acetyl Cysteine (NAC; antioxidant)^28^, alpha smooth muscle actin (α-SMA) Ab (Sigma- Aldrich, St Louis, MO), Amplex Red (Thermo Fisher Scientific, Waltham, MA, USA), Fibronectin (FN) Ab (Abcam, Cambridge, MA), Myocardin-Related Transcription Factor-A (MRTF-A) Ab (Santa Cruz Biotechnology Santa Cruz, CA), glyceraldehyde-3-phosphate dehydrogenase (GAPDH) Ab (Fitzgerald, Acton, MA), all secondary Abs (Jackson ImmunoResearch, West Grove, PA), non-targeting small interfering RNA (siRNA) and specific siRNA for TRPV4 and NOX4 (Dharmacon, Lafayette, CO), siLentFect lipid reagent (Bio-Rad, Hercules, CA), transcriptor first strand cDNA synthesis kit and light cycler 480 SYBR Green I Master Mix (Roche, Indianapolis, IN).

### D. farinae induced airway remodeling

House dust mite (D*. farinae*) protein extract was purchased from Greer Laboratories (Lenoir, NC). *D. farinae* extract was resuspended in phosphate buffer saline (PBS) (Corning, NY) at a concentration of 2 mg/mL. Following isofluorane, 6–8 week old WT and TRPV4 KO mice received *D.farinae (*25 μg/animal) or saline intranasally, 3 times a week for 5 weeks. Mice were euthanized 24 h after the last intranasal instillation. RNA was isolated from the lungs and real time quantitative PCR (qPCR) was performed to analyze NOX4 transcript.

Broncho alveolar lavage (BAL) fluid was collected and cellular content was analyzed. After BAL fluid was collected, total cell number was determined by trypan blue staining. Differential cell count was obtained using Diff Quik Stain Set (Siemens, Newark, DE). Briefly, BAL fluid was cytospun (Cytospin, Thermo Fisher Scientific, Waltham, MA) onto glass slides, and stained with Diff Quik Stain Set according to the manufacturer’s protocol. A minimum of 300 cells were counted, and identified as macrophages, lymphocytes, or polymorphonuclear leukocytes (PMNs- eosinophils and neutrophils) based on morphological criteria. Lungs from mice were embedded in paraffin, sectioned, and stained for periodic Acid Schiff (PAS) to visualize goblet cells.

### Cell culture

Validated primary normal human LF (NHLF) (CC-2512) and diseased (asthma; Male, 27 years old, diagnosed at age 7 and on medication (Proventil, Albuterol)) human LF (DHLF) (00194912) were obtained from LONZA (Walkersville, MD). Cells from same passage for both NHLF and DHLF were cultured in Dulbecco’s Modified Eagle’s Medium (DMEM, Corning) supplemented with 10% fetal bovine serum (FBS, Atlanta Biologicals, Norcross, GA), 100 units/ml penicillin-streptomycin, 2 mM L-glutamine (Invitrogen) and maintained at 37 °C in a humidified 5% CO_2_ environment. Both NHLF and DHLF were used below passage 8 and comparable passages were used in different experiments. Primary mouse LF were isolated from 10-week-old WT and TRPV4 KO mice. Briefly, lungs were removed aseptically, minced into ~1 mm pieces and incubated with agitation in 0.2% trypsin and 0.2% collagenase in DMEM medium supplemented with 10% fetal bovine serum, 100 units/ml penicillin-streptomycin, 2 mM L-glutamine for 30 minutes. The resulting cell suspension was filtered with a cell strainer (40 μm) (BD Biosciences, San Jose, CA) and centrifuged at 500 x g for 5 minutes. Cell pellet was washed, re-suspended and cultured in DMEM medium (10% FBS, 100 units/ml penicillin-streptomycin and 2 mM L-glutamine) in 25 cm^2^ tissue culture flasks and maintained at 37 °C in a humidified 5% CO_2_ environment.

### Cell activation and treatment

NHLF and/or DHLF cells were pre-treated with inhibitors RN1734 (30 μM), LY294002 (50 μM), NAC (5, 10 mM), DPI (0.5, 1 µM), EHT (30–100 µM) for 30 minutes followed by stimulation with TGFβ1 (2 ng/mL for 48 h) to analyze the expression of α-SMA, FN, PAI-1, and MRTF-A expression. Transfection of isoform-specific siRNA smart pool constructs against TRPV4 from Dharmacon (20 nmol/L) and NOX4 from Qiagen (100 nM) were carried out with siLentFect transfection reagent for 48 h, according to the manufacturer’s protocol.

### Determination of extracellular H_2_O_2_

Extracellular H_2_O_2_ levels were determined by using Amplex Red (Molecular Probes) according to manufacturer’s protocol. Briefly, NHLF (7.5 × 10^4^) were treated with and without TGFβ1 (2 ng/mL) in the presence or absence of inhibitors mentioned above. After treatment, cells were washed twice in Krebs-Ringer phosphate glucose buffer (KRPG) and incubated for 30 minutes with Amplex Red reagent (50 µM) and 0.1 U/mL HRP in KRPG. Fluorescence was measured with excitation and emission at 530 and 590 nm respectively using fluorescence spectrophotometer (Hitachi F-4500) and the data is presented as relative fluorescence units (RFU).

### Cell lysates and immunoblotting

After stimulation with the respective agonists, NHLF (7.5 × 10^4^) were lysed with lysis buffer (BD Bioscience, San Jose, CA) supplemented with protease inhibitor cocktail (Roche) and phosphatase inhibitor cocktail (Pierce, Rockford, IL). Immunoblotting was performed as previously described^29^. Briefly, lysates were subjected to 4–12% SDS-PAGE and transferred to polyvinylidene difluoride membranes. The membranes were blocked and incubated with Abs against α-SMA, FN, MRTF-A and PAI-1 in 1% Tris buffer saline (TBS) 5% dry milk, 0.1% Tween-20) (1:1000) overnight at 4 °C on shaker, and then with secondary Ab. Bands were visualized with enhanced chemiluminiscence (ECL) and protein bands were visualized using an imager (ProteinSimple, San Jose, CA). The blots were stripped and re-probed with Glyceraldehyde 3-phosphate dehydrogenase (GAPDH) Ab (1:25000). Densitometric analysis of the bands on the blots were quantified by AlphaView software (version 3.4, ProteinSimple).

Real-time quantitative PCR

The expressions of α-SMA, collagen 1A1, FN, MRTF-A, SM22, and PAI-1 transcripts were determined with qPCR performed on Light cycler 480 (Roche). Total RNA was isolated from NHLF and DHLF after respective treatments with an E.Z.N.A. Total RNA kit 1 (Omega Bio-Tek, Norcross, Georgia). RNA from tissues was extracted by Trizol-chloroform and was treated with RNase-free DNase (Invitrogen). DNAse contamination was removed using DNA-free DNA Removal Kit (Invitrogen) based on the manufacturer’s instructions. cDNA was synthesized using cDNA synthesis kit from Roche. qPCR was performed using primers mentioned below. Levels of respective genes relative to the GAPDH were analyzed and the ∆ΔCT values were calculated and expressed as relative expression or fold change compared to control. Quality of the RNA, primers and real time PCR reaction was validated using proper controls like no RT control and water control. Real time PCR for each sample was performed in at least triplicate and repeated in three different experiments.

#### Primers

***h NOX4*** Qiagen (Hilden, Germany) catalog no. PPH06078A-200

***h α-SMA***

F: 5′-CGGGACATCAAGGAGAAACT-3′

R: 5′-CCATCAGGCAACTCGTAACT-3′

***h COLLAGEN 1 A1***

F: 5′-CGATGGATTCCAGTTCGAGTATG-3′ R: 5′-CTTGCAGTGGTAGGTGATGTT-3′

***h FN***

F: 5′-ACAACACCGAGGTGACTGAGAC-3′

R: 5′-GGACACAACGATGCTTCCTGAG-3′

***h SM22***

F: 5′-TGGAGATCCCAACTGGTTTAT-3′

R: 5′-CCCATCTGAAGGCCAATG -3′

***h TRPV4***

F: 5′-TCACTCTCACCGCCTACTACC A-3′

R: 5′-CCCAGTGAAGAGCGTAATGACC-3′

***h NOX1***

F: 5′-GGTTTTACCGCTCCCAGCAGAA-3′

R: 5′-CTTCCATGCTGAAGCCACGCTT-3′

***h NOX2***

F: 5′-CTCTGAACTTGGAGACAGGCAAA-3′

R: 5′-CACAGCGTGATGACAACTCCAG-3′

***h NOX3***

F: 5′-CCTGGAAACACGGATGAGTGAG-3′

R: 5′-CCTCCCATAGAAGGTCTTCTGC-3′

***h NOX5***

F: 5′-CCACCATTGCTCGCTATGAGTG-3′

R: 5′-GCCTTGAAGGACTCATACAGCC-3′

***h GAPDH***

F: 5′-TGCACCACCAACTGCTTAGC-3′

R: 5′-GGCATGGACTGTGGTCATGAG-3′

***m NOX4***

F: 5′-CGGGATTTGCTACTGCCTCCAT-3′

R: 5′-GTGACTCCTCAAATGGGCTTCC-3′

***m α-SMA***

F: 5′-CCCAGACATCAGGGAGTAATG -3′

R: 5′-GCCGTGTTCTATCGGATACTT -3′

***m GAPDH***

F: 5′-CTCCCACTCTTCCACCTTCG-3′

R: 5′-CCACCACCCTGTTGCTGTAG-3′

### siRNA transfection

NHLF cells were transfected with siGENOME SMART pool (a mix of 4 pre-made siRNA; Dharmacon, Lafayette, CO) of 20 nM TRPV4 specific siRNA to block TRPV4, 100 nM NOX4-specific siRNA (to block NOX4) or non-specific siRNA (negative control). The sequence information for siRNA oligos is given below. Transfection was carried out with siLentFect transfection reagent (Bio-Rad) according to manufacturer’s instructions. Post transfection, cells were treated as mentioned previously^19^.

h TRPV4- siGENOME SMART pool (Cat# M-004195-00-0005)

Target Sequence 1: GAACCCGUGUGCCAACAUG

Target Sequence 2: CAACCGGCCUAUCCUCUUU

Target Sequence 3: GACCAAAUCUGCGCAUGAA

Target Sequence 4: GCACACCGCCGUACCCUUA

h NOX4- siGENOME SMART pool (Cat# M-010194-00)

Target Sequence 1: GAAUUACAGUGAAGACUUU

Target Sequence 2: CAGGAGGGCUGCUGAAGUA

Target Sequence 3: GGGCUAGGAUUGUGUCUAA

Target Sequence 4: GAUCACAGCCUCUACAUAU

Non-Targeting- siGENOME Pool#2 siRNA (Cat# D-001206-14-05)

### Immunofluorescence

After respective treatment, NHLF were fixed with 4% paraformaldehyde/PBS solution, permeabilized in 0.5% Triton X-100 for 15 min, washed twice in PBS, blocked with 10% FBS containing medium. Thereafter, cells were stained for 1 h with α-SMA antibody, followed by PBS washes and incubation with Alexa Fluor 594 goat anti-mouse secondary antibody for 45 minutes. Following secondary antibody, cells are mounted in fluorescence mounting medium containing DAPI. Images were obtained using EVOS fluorescence microscope.

### Statistical analysis

Western blots presented are representative of three experiments performed and data are expressed as means ± SEM from at least three experiments except where otherwise indicated. Significance was determined using one-way analysis of variance (ANOVA) and comparisons between the groups were determined by Tukey’s multiple comparisons test (GraphPad Prism). *P < 0.05, **P < 0.01, ***P < 0.001.

## Results

### TGFβ1-induced ROS mediate NHLF differentiation

**Figure 1:**
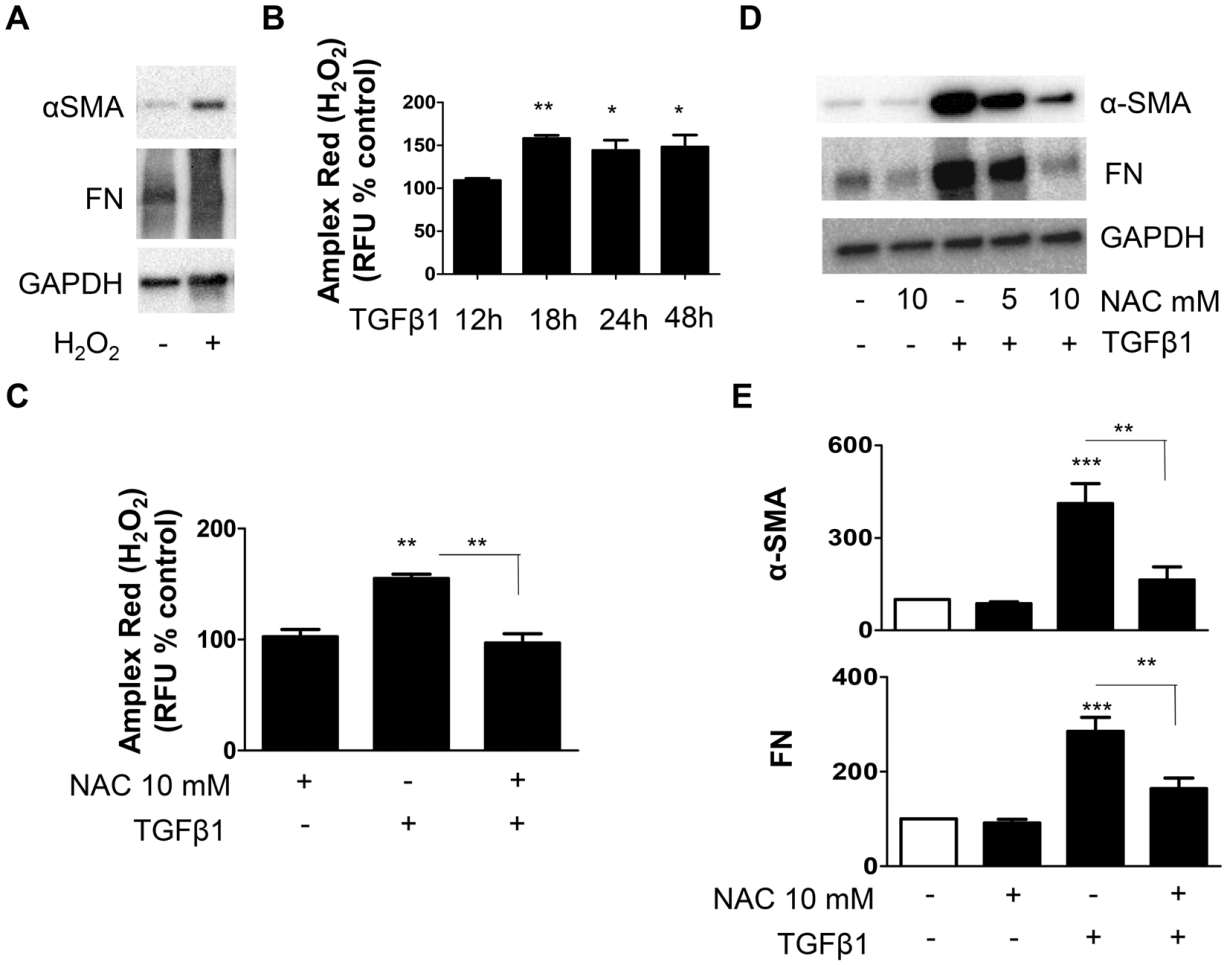
TGFβ1- induced NHLF differentiation is mediated through ROS. (**A**) NHLF were treated with H_2_O_2_ (50 µM) for 48 h, and SDS-PAGE immunoblotting was performed on cell lysates using Abs specific for α-SMA and FN proteins. Thereafter, the blots were stripped and reprobed for GAPDH. Representative blots are from a single experiment of three performed. (**B**) NHLF were treated with TGFβ1 (2 ng/mL) for the indicated time points, and ROS (H_2_O_2_ generation) was measured using Amplex Red assay. After treatment, cells were washed twice in KRPG and incubated for 30 minutes with Amplex Red reagent (50 µM) and 0.1 U/mL HRP in KRPG. Fluorescence was measured with excitation and emission at 530 and 590 nm respectively and expressed as relative fluorescence units (RFU). H_2_O_2_ generated in response to TGFβ1 is expressed as percentage of fold change in RFU compared to control. Data are means + SEM from three independent experiments. For (**C-E**), NHLF were pre-treated for 30 minutes in the presence or absence of indicated concentration of NAC (antioxidant), followed by treatment with TGFβ1 (2 ng/mL; 48 h). *C)* ROS (H_2_O_2_) generation was measured using Amplex Red assay and expressed as percentage of control RFU. Data are means + SEM from three independent experiments. (**D**) SDS-PAGE immunoblotting was performed on cell lysates using Abs specific for α-SMA and FN proteins. Representative blots are from a single experiment of five performed. (**E**) Represents quantitative densitometric analysis of indicated proteins from (**D**) using AlphaView software and expressed as a percentage of control cells. Results are means + SEM from five independent experiments *P < 0.05; **P < 0.01; ***P < 0.001.

To determine if oxidative stress is involved in the differentiation of fibroblasts to myofibroblasts, we stimulated NHLF with H_2_O_2_ and analyzed the expression of α-SMA and FN proteins by western blotting. We found that stimulation of NHLF with H_2_O_2_ resulted in a significant up-regulation of both α-SMA and FN proteins (Fig. 1A). Since TGFβ1 is the main driver for fibroblast differentiation, we analyzed if TGFβ1 could enhance ROS (H_2_O_2_) using the Amplex Red assay. We observed an increase in ROS generation in response to TGFβ1 at 18 h and plateaued (Fig. 1B). We next asked if this ROS generated by TGFβ1 is required for NHLF differentiation. To address this, we pre-treated NHLF with an antioxidant, NAC, stimulated with TGFβ1, and analyzed the expression of ROS, α-SMA and FN proteins. NAC pre-treatment significantly reduced TGFβ1-enhanced ROS (Fig. 1C), α-SMA and FN protein expression (Fig. 1D,E), as well as the incorporation of α-SMA into the stress fibers (supplemental Fig. 1A), suggesting that oxidative stress plays an important role in TGFβ1-induced differentiation of NHLF.

### NOX4 is required for TGFβ1 induced NHLF differentiation

**Figure 2:**
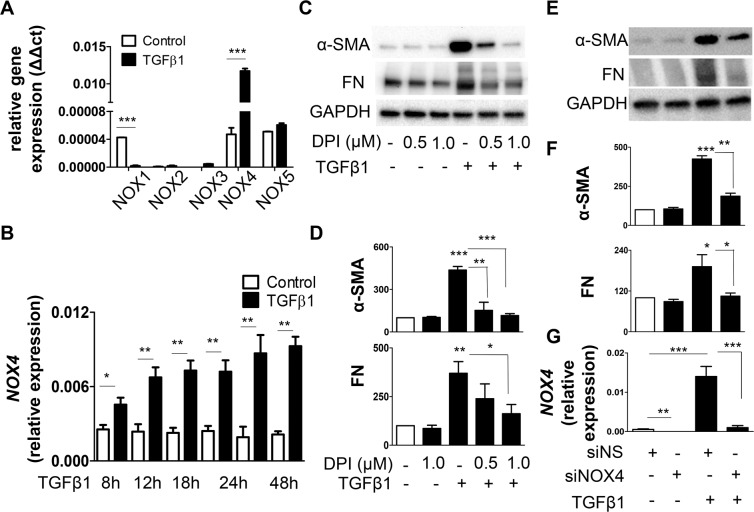
NOX4 is essential for TGFβ1-induced NHLF differentiation. (**A**) qPCR showing relative levels (ΔΔct compared to GAPDH) of NOX1-NOX5 transcript expression by NHLF stimulated with TGFβ1 (2 ng/mL; 48 h). Data are means + SEM from three experiments. (**B**) NHLF were treated with TGFβ1 (2 ng/mL) for the indicated time points and qPCR analysis of NOX4 transcript expression was performed. Data are means + SEM from three experiments. (**C**) NHLF were pre-treated (30 minutes) in the presence or absence of indicated concentrations of DPI (NOX4 inhibitor), followed by treatment with TGFβ1 (2 ng/mL; 48 h). SDS-PAGE immunoblotting was performed on cell lysates using Abs specific for α-SMA and FN proteins. Thereafter, the blots were stripped and re-probed for GAPDH. Representative blots are from a single experiment of three performed. (**D**) Represents quantitative densitometric analysis of indicated proteins from (**C**) using AlphaView software and expressed as a percentage of control cells. Results are means + SEM from three independent experiments. (**E–G**) NOX4 protein was knocked down in NHLF by transfecting them with siRNA against NOX4 (100 nM). NHLF transfected with nonspecific (NS) siRNA were used as control. Twenty four hours after transfection, NHLF were treated with TGFβ1 (2 ng/mL; 48 h). (**E**) α-SMA and FN protein levels were analyzed using immunoblotting, following which the blots were stripped and re-probed for GAPDH (**F**) shows quantitative densitometric analysis of indicated proteins from (**E**) using AlphaView software and expressed as a percentage of control cells. (**G**) NOX4 transcript was analyzed by qPCR. Results are means + SEM from three independent experiments. *P < 0.05; **P < 0.01; ***P < 0.001.

We next asked if TGFβ1 mediates fibroblast differentiation through the up-regulation of NOX. We treated NHLF with TGFβ1 for 48 h and then evaluated the expression levels of NOX1-5. We found that TGFβ1 stimulation resulted in significant increase in *NOX4* transcript and reduction in *NOX1* but had no effect on the expression of NOXs 2, 3 and 5 (Fig. 2A). Further, TGFβ1 promoted a time-dependent upregulation of NOX4, which started at 8 h and plateaued (Fig. 2B). Next, to determine if NOX mediates TGFβ1-induced differentiation, we pre-treated NHLF with a general NOX inhibitor, DPI and examined the levels of α-SMA and FN proteins. DPI pre-treatment significantly inhibited TGFβ1-induced α-SMA and FN protein expression (Fig. 2C,D) as well as the incorporation of α-SMA into the stress fibers (supplemental Fig. 1B). To determine the specific role of NOX4 in TGFβ1-induced differentiation, we knocked down *NOX4* in NHLF by NOX4-specific siRNA and analyzed TGFβ1-mediated NHLF differentiation. We found that NOX4-specific siRNA significantly down regulated both basal and TGFβ1-mediated *NOX4* expression (Fig. 2G) and NHLF differentiation as evidenced by reduced α-SMA and FN levels (Fig. 2E,F). NOX4 specific siRNA, but not non-specific siRNA, reduced NOX4 expression, confirming the specific down regulation of NOX4 by the NOX4 siRNA oligos used in the experiment (Fig. 2G).

### NOX4 mediates TGFβ1-induced MRTF-A activation, fibrotic gene expression, and inhibits matrix degradation

**Figure 3:**
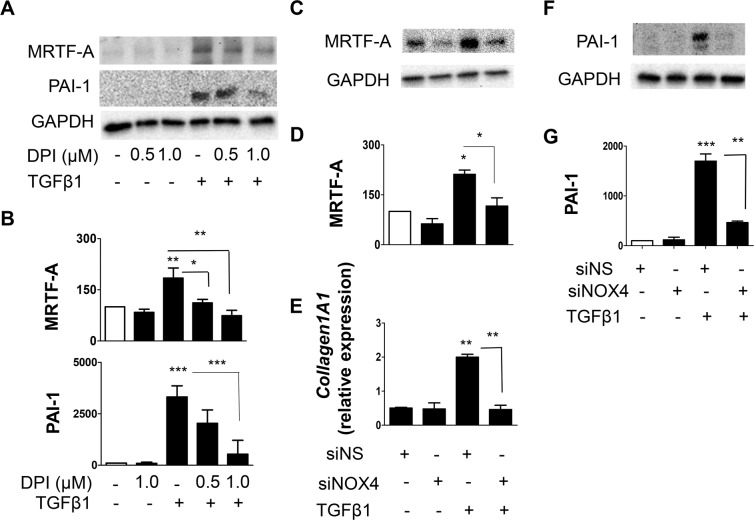
NOX4 regulates TGFβ1-induced matrix accumulation by enhancing fibrotic gene expression and inhibiting matrix degradation by PAI-1 activation. (**A,B**) NHLF were pre-treated (30 minutes) in the presence or absence of indicated concentrations of DPI (NOX4 inhibitor), followed by treatment with TGFβ1 (2 ng/mL; 48 h). (**A**) SDS-PAGE immunoblotting was performed on cell lysates using Abs specific for MRTF-A and PAI-1 proteins. Thereafter, the blots were stripped and re-probed for GAPDH. Representative blots are from a single experiment of three performed. (**B**) Represents quantitative densitometric analysis of indicated proteins from (**A**) using AlphaView software and expressed as a percentage of control cells. Results are means + SEM from three independent experiments. (**C–G**) NOX4 protein was knocked down in NHLF by transfecting them with siRNA against NOX4 (100 nM). NHLF transfected with nonspecific (NS) siRNA were used as control. Twenty four hours after transfection, NHLF were treated with TGFβ1 (2 ng/mL; 48 h). (**C**) MRTF-A protein was analyzed using immunoblotting, following which the blots were stripped and re-probed for GAPDH (**D**) shows quantitative densitometric analysis of MRTF-A using AlphaView software and expressed as a percentage of control cells. (**E**) Collagen1A1 transcript was analyzed by qPCR. Results are means + SEM from three independent experiments. (**F**) PAI-1 protein (responsible inhibition of for matrix degradation) was analyzed using immunoblotting, following which the blots were stripped and re-probed for GAPDH (**G**) shows quantitative densitometric analysis of PAI-1 using AlphaView software and expressed as a percentage of control cells. Results are means + SEM from three independent experiments. *P < 0.05; **P < 0.01; ***P < 0.001.

Fibrotic gene expression is regulated by serum responsive factor (SRF) and its co-activators of the myocardin family^30^. MRTF-A is a mechanosensitive transcription factor, which is known to be activated in response to stress fiber formation via Rho^31^ and activates fibrotic gene expression. We have previously shown that TGFβ1 enhanced the expression and translocation of MRTF-A to the nucleus in LF^19^. Since NOX4 is involved in TGFβ1-mediated NHLF differentiation, we investigated if NOX4 regulates TGFβ1-induced MRTF-A, and expression of fibrotic genes. TGFβ1 stimulation significantly enhanced MRTF-A protein expression, which is attenuated by NAC (supplemental Fig. 2A,B), DPI (Fig. 3A,B), and NOX4 siRNA (Fig. 3C,D). Further, we found that TGFβ1 induced higher expression of fibrotic genes including collagen1A1 (Fig. 3E), SM22, and FN (supplemental Fig. 3A,B), which was attenuated by NOX4 siRNA. Matrix accumulation is a balance between matrix synthesis and degradation. Since our results indicated that NOX4 enhances fibrotic gene expression and matrix synthesis in response to TGFβ1, we further speculated if NOX4 also can regulate matrix degradation. Plasmin, which is involved in the degradation of ECM components is activated from plasminogen by tissue-type plasminogen activator (t-PA) or urokinase-type PA (u-PA), and plasminogen activator inhibitor-1 (PAI-1) is a major inhibitor of both t-PA and u-PA^32^. Since we demonstrated earlier that TGFβ1 increased the expression of PAI-1 at both the transcript and protein levels^19^, we asked if NOX4 can also regulate PAI-1 expression. Inhibition of NOX4 either pharmacologically by NAC (supplemental Fig. 2C,D), DPI (Fig. 3A,B), or by NOX4 siRNA (Fig. 3F,G) significantly attenuated PAI-1 protein expression.

### TRPV4 integrates TGFβ1 and NOX4 signals during fibroblast differentiation

**Figure 4:**
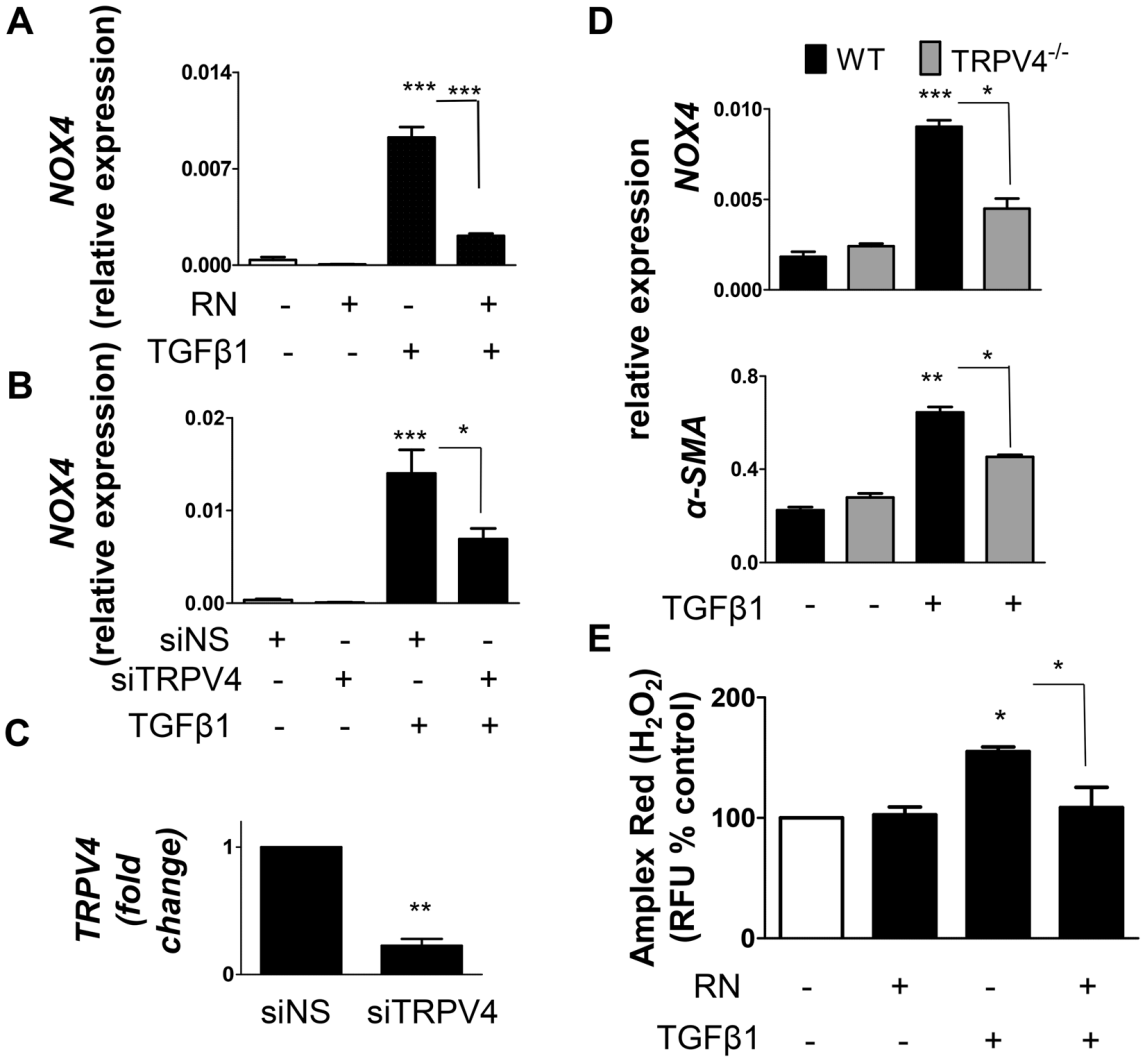
TRPV4 regulates TGFβ1-mediated NHLF differentiation through NOX4. (**A**) NHLF were pre-treated (30 minutes) in the presence or absence of a TRPV4 inhibitor, RN1734 (30 μM) followed by treatment with TGFβ1 (2 ng/mL; 48 h). Graph represents qPCR analysis of NOX4 transcript expression. Data are means + SEM from three experiments. (**B,C**) TRPV4 protein was knocked down in NHLF by transfecting them with siRNA against TRPV4 (20 nM). NHLF transfected with nonspecific (NS) siRNA were used as control. Twenty four hours after transfection, NHLF were treated with TGFβ1 (2 ng/mL; 48 h). (**B**) NOX4 transcript expression was analyzed using qPCR and expressed as ΔΔct. (**C**) TRPV4 transcript expression was analyzed using qPCR. The expression in TRPV4 siRNA treated cells is compared to NS siRNA treated cells and is expressed as fold change compared to NS siRNA treatment, where NS siRNA treatment is set to 1. (**D**) Mouse LF isolated from WT and TRPV4 KO mice were treated with TGFβ1 (2 ng/ml) for 48 h. The expression of NOX4 and α-SMA were analyzed by qPCR and expressed as ΔΔct. (**E**) NHLF were pre-treated (30 minutes) in the presence or absence of a TRPV4 inhibitor, RN1734 (30 μM) followed by treatment with TGFβ1 (2 ng/mL; 48 h). ROS (H_2_O_2_ generation) was measured using Amplex Red assay. After treatment, cells were washed twice in KRPG and incubated for 30 minutes with Amplex Red reagent (50 µM) and 0.1 U/mL HRP in KRPG. Fluorescence is measured expressed as relative fluorescence units (RFU) compared to control. Data are means + SEM from three independent experiments. *P < 0.05; **P < 0.01; ***P < 0.001.

We have previously shown that TRPV4 is critical for TGFβ1-induced NHLF differentiation^19^, while our current results implicate a strong role for NOX4 in TGFβ1-mediated effects. Therefore, we examined if there is an interaction between TRPV4 and NOX4. To achieve this, we pre-treated NHLF with either TRPV4 inhibitor RN or TRPV4 siRNA, stimulated with TGFβ1 and analyzed NOX4 expression. We observed that TRPV4 inhibition by pharmacological inhibitor RN (Fig. 4A) or siRNA downregulation (Fig. 4B) significantly attenuated TGFβ1-mediated NOX4 expression. TRPV4 siRNA, but not non-specific siRNA, induced TRPV4 inhibition, confirming the specific down regulation of TRPV4 by TRPV4 siRNA oligos (Fig. 4C). While TGFβ1 treatment enhanced NOX4 and α-SMA expression in WT fibroblasts, this response was completely attenuated in TRPV4 KO fibroblasts (Fig. 4D). Further, we found that TRPV4 inhibitor RN significantly inhibited TGFβ1-induced NOX4 activity (ROS generation; Fig. 4E). Taken together, these results demonstrate that TRPV4 regulates NOX4 expression and activity downstream of TGFβ1.

### PI3K and Rac regulate TGFβ1-mediated NOX4 expression and function

**Figure 5:**
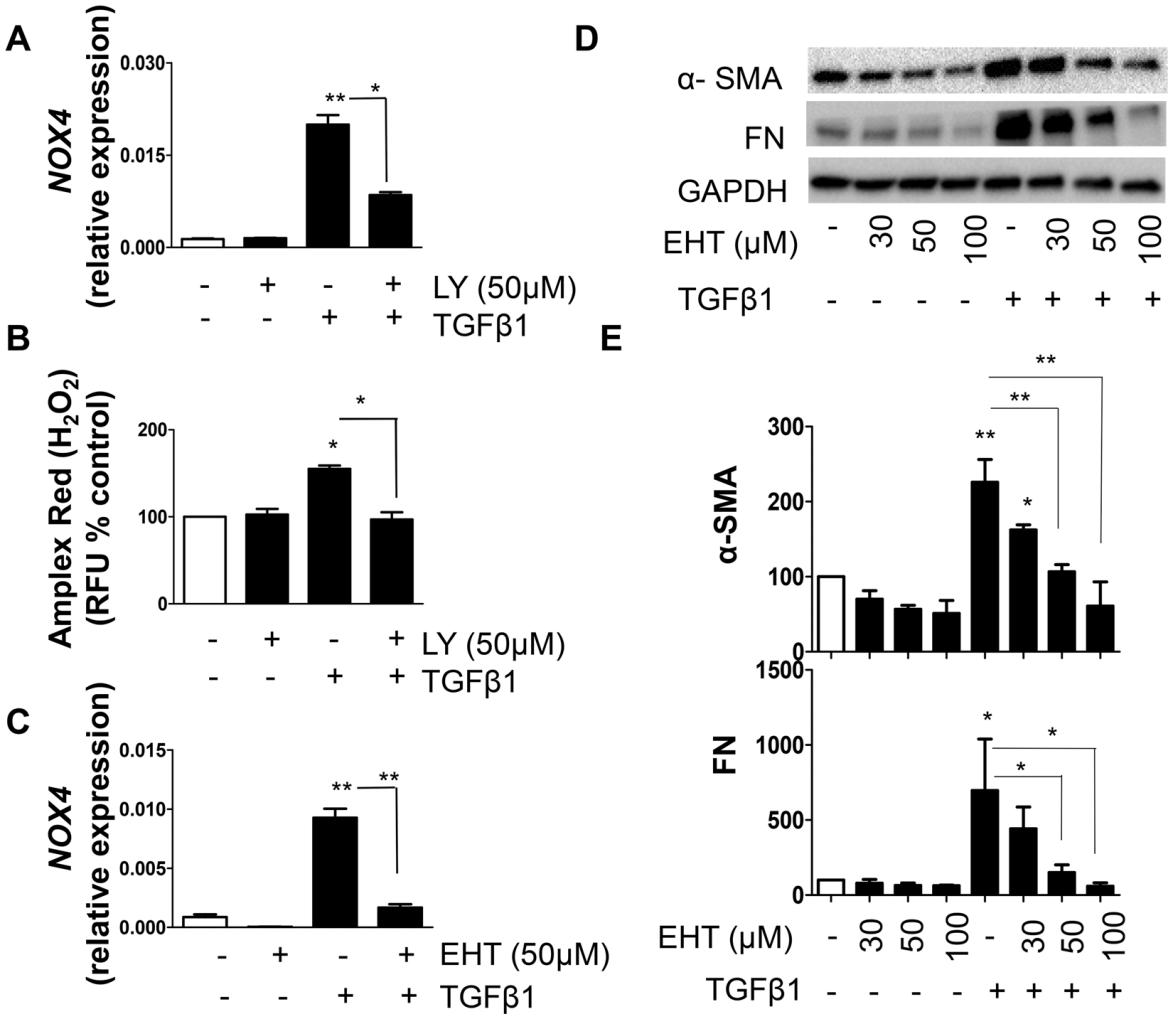
PI3K and Rac modulate TGFβ1-mediated NOX4 expression. (**A,B**) NHLF were pre-treated (30 minutes) in the presence or absence of a PI3K inhibitor, LY294002 (50 µM) followed by treatment with TGFβ1 (2 ng/mL; 48 h). (**A**) qPCR analysis of NOX4 transcript expression expressed as ΔΔct. Data are means + SEM from three experiments. (**B**) ROS (H_2_O_2_ generation) was measured using Amplex Red assay. After treatment, cells were washed twice in KRPG and incubated for 30 minutes with Amplex Red reagent (50 µM) and 0.1 U/mL HRP in KRPG. Fluorescence is measured expressed as relative fluorescence units (RFU) compared to control. Data are means + SEM from three independent experiments. (**C–E**) NHLF were stimulated with TGFβ1 (2 ng/mL; 48 h) in the presence or absence of indicated concentrations of a rac inhibitor EHT, and (**C**) NOX4 transcript was measured using qPCR and expressed as ΔΔct. (**D**) SDS-PAGE immunoblotting was performed on cell lysates using Abs specific for α-SMA and FN proteins after which, the blots were stripped and re-probed for GAPDH. Representative blots are from a single experiment of three performed. (**E**) Represents quantitative densitometric analysis of indicated proteins from (**D**) using AlphaView software and expressed as a percentage of control cells. Results are means + SEM from three independent experiments. *P < 0.05; **P < 0.01.

Next, we investigated the mechanism by which TGFβ1 /TRPV4 axis regulates NOX4. We have demonstrated earlier that TGFβ1 activates PI3K in NHLF and PI3K is required for the translocation of TRPV4 to the membrane and NHLF differentiation^19^. We found that pre-treatment with PI3K inhibitor LY significantly inhibited TGFβ1-mediated NOX4 expression and ROS generation (Fig. 5A,B). Among several PI3K downstream targets, Rac-1 has been established to transduce PI3K signals to generate ROS in fibroblasts^33^. Therefore, we pre-treated NHLF with Rac-1 inhibitor EHT, stimulated with TGFβ1, and analyzed NOX4 and fibrotic gene expression. EHT pre-treatment completely inhibited TGFβ1-mediated NOX4 expression (Fig. 5C), H_2_O_2_ generation (supplemental Fig. 3C) as well as α-SMA and FN proteins (Fig. 5D,E).

### TRPV4/NOX4 axis mediates airway remodeling in asthma

**Figure 6:**
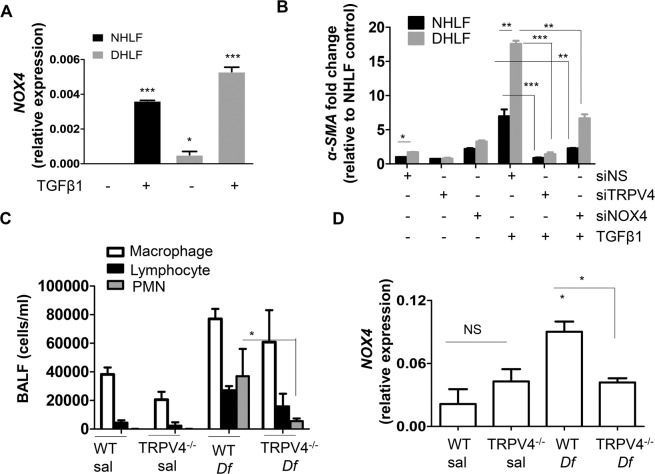
TRPV4/NOX4 axis plays a vital role in airway remodeling in asthma. (**A**) Differential responses of NHLF and DHLFs to TGFβ1. NHLF and DHLF were stimulated with TGFβ1 (2 ng/mL; 48 h), and (**A**) qPCR analysis of NOX4 transcript expression expressed as ΔΔct. (**B**) TRPV4 protein and NOX4 protein was knocked down in NHLF by transfecting them with siRNA against TRPV4 (20 nM) or NOX4 (100 nM). NHLF transfected with nonspecific (NS) siRNA were used as a control. Twenty four hours after transfection, NHLF were treated with TGFβ1 (2 ng/mL; 48 h) and NOX4 transcript was analyzed by qPCR and expressed as fold change relative to NHLF control. (**C,D**) WT and TRPV4 KO mice were treated with saline or *D. farinae* (25 mg/animal) intranasally 3 times a week for 5 weeks. Mice were euthanized 24 h after the final challenge and c) differential cell counts from the BAL fluid was analyzed. (**D**) Expression of NOX4 normalized to GAPDH was determined by qPCR of whole lung RNA extracted from the indicated groups. Results are means ± SEM from at least 3 independent experiments. *P < 0.05; **P < 0.01; ***P < 0.001.

To understand the significance of the TRPV4-NOX pathway in fibroblast differentiation in asthma, we took advantage of fibroblasts isolated from normal (NHLF) and from patient suffering from asthma exacerbation (DHLF) and analyzed NOX4 expression in both cell types. Interestingly, we found NOX4 expression is higher in DHLF compared to NHLF (Fig. 6A). Further, TGFβ1 treatment significantly increased NOX4 expression and differentiation in DHLF compared to NHLF (Fig. 6B). Importantly, siRNA knockdown of either NOX4 or TRPV4, in NHLF and DHLF, significantly attenuated TGFβ1-mediated α-SMA expression (Fig. 6B). To determine the pathophysiological significance of TRPV4/NOX4 axis in asthma, WT and TRPV4 KO mice were subjected to intranasal inhalation of either saline or *D. farinae* antigen (25 μg), three times a week for five weeks and analyzed immune cells in BAL fluid, goblet cells and NOX4 transcript expression in lung. We observed increased recruitment of lymphocytes and polymorphonuclear leukocytes (PMN) to the lung (Fig. 6C), goblet cell metaplasia (supplemental Fig. 4) only in WT mice, but not TRPV4 KO mice. NOX4 expression was significantly upregulated in response to *D. farinae* in WT mice compared to saline treated mice (Fig. 6D). In contrast, there was no change in NOX4 expression in response to *D. farina*e in TRPV4 KO mice (Fig. 6D).

## Discussion

Although fibroblast differentiation is critical in physiological airway remodeling, uncontrolled differentiation of fibroblasts to myofibroblasts can lead to lung fibrosis. In fact, lung biopsy samples from several asthmatics reveal enhanced fibroblast numbers compared to normal subjects, which correlated to the thickening of the lamina reticularis^3,34^. However, the precise molecular mechanism(s) underlying fibroblasts differentiation are still elusive. In parallel to the extensive lung remodeling observed in asthmatic airways, enhanced expression of TGFβ1 was also observed^35^. Soluble factor, TGFβ1, has been identified as a master regulator for fibroblast to myofibroblast differentiation^10–12^ *in vitro* and in tissue fibrosis of many organs^36,37^ via canonical and non-canonical pathways. TGFβ1 signals through TGFβ1 receptor types I and II via SMAD-dependent or SMAD-independent pathways like PI3K, p38 MAPK, focal adhesion kinases, and Rho GTPases to drive myofibroblast differentiation. Although TGFβ1 is a major driver of myofibroblast differentiation, targeting it is not an option to prevent aberrant remodeling due to its pleiotropic nature. This makes it imperative to understand signals downstream of TGFβ for the therapy of fibrotic disorders, including airway remodeling. Apart from soluble factors, an abnormal mechanical environment has been predicted to modify the composition of the ECM in airways^14^. Interestingly, structural remodeling of the airways has been found in children with recurrent wheezing, regardless of their atopic status^38^. TRPV4, a mechanosenstive ion channel has been found to regulate pulmonary function in ventilator-induced lung injury^39^ and airway remodeling^19^. Although TRPV4 is activated by physical and chemical stimuli (temperature, hypo tonicity, phorbol esters, endocannabinoids, arachidonic acid (AA) and its metabolites, epoxy eicosatrienoic acids (EETs)^40–43^, we and others recently demonstrated that TRPV4 is also activated by TGFβ^18,19,23^ and play a vital role in lung remodeling. Intriguingly, TRPV4 gene polymorphisms have been shown to influence the development of osmotic airway hyperresponsiveness in patients with bronchial asthma^16^. However, the mechanism through which TRPV4 is activated or whether and how TRPV4 facilitates fibroblast differentiation and matrix remodeling, specifically in asthma, is still elusive. Besides soluble and mechanical signals, enhanced ROS production is well documented in asthma, and the airways of asthmatics produce more ROS than subjects with healthy lungs^44–46^. TGFβ1 is known to promote myofibroblast differentiation through ROS^47^, and high levels of superoxides are generated by NOX^20^. Specifically, NOX4 isoform that is transcriptionally regulated and constitutively active was found to mediate contractility, matrix production, and α-SMA expression. Although NHLF express NOX1, 4 and 5 transcripts, we found significant up-regulation of only NOX4 in response to TGFβ1. TGFβ1 has been shown to induce NOX4 expression in lung mesenchymal cells via SMAD-3-dependent pathway and NOX4-dependent ROS generation is required for TGFβ1- induced myofibroblast differentiation, ECM production and contractility^48^. Since both mechanical factors and ROS are crucial players in regulating myofibroblast differentiation and airway remodeling, we explored if there exits an interaction between TRPV4 and NOX downstream of TGFβ1. Our results demonstrated that TGFβ1 enhances ROS generation and NOX4 expression. Unfortunately, we could not validate NOX4 expression at protein level due to the non-specific NOX4 Abs currently available. Since enhanced ECM build-up is a characteristic of lung remodeling because of fibroblast differentiation, we asked if NOX4 contributes to increase in matrix synthesis, or reduction in matrix degradation, or both. Fibrotic gene expression is regulated by the co-activators of the myocardin family. Upon activation, MRTF-A translocates to the nucleus, associates with SRF CC(A/T)6GG (CArG box) present in the promoter of target genes, including α-SMA. Interestingly, mechanical forces are known to stimulate translocation of MRTF-A transcription factor into the nucleus^30^. NOX4 has been shown earlier to regulate fibrotic gene expression and matrix synthesis through MRTF-A in kidney epithelial cells^49^. In accordance, we found that NOX4 mediates TGFβ1-induced fibrotic gene expression (collagen1A1, α-SMA, FN, and SM-22) via activation of MRTF-A during fibroblast differentiation. Further, we found that NOX4 also contributes to enhanced ECM by modulating matrix degradation (by increasing PAI-1 expression). Although ROS have been implicated in TRPV4-induced neuronal signaling^50^, the TRPV4-NOX interaction has not been reported thus far. Intriguingly, we found that knocking down TRPV4 attenuated NOX4 induction by TGFβ1, suggesting that TRPV4 acts upstream of NOX4. Further, PI3K inhibitor LY, which significantly inhibited TRPV4 translocation to the membrane^19^, also attenuated NOX4 expression and activity, suggesting that both TRPV4 and NOX4 are downstream of PI3K. We further explored how PI3K regulates NOX4. In fibroblasts, PI3K mediates PDGF-dependent ROS production via Rac-1^33^. Therefore, we wondered if PI3K mediates ROS production via Rac-1 downstream of TGFβ1 as well. Interestingly, Rac inhibition significantly inhibited TGFβ1-mediated NOX4 expression, activity, and fibrotic gene expression. NOX4 has been implicated in relaying pro-fibrotic responses to TGFβ^47^, and pharmacological inhibition or knockdown of the NOX4 transcript has been shown to suppress matrix synthesis and experimentally induce lung fibrosis in mice^48^. However, the role of NOX4 in asthma is still elusive. Our results demonstrated that at basal levels, LF isolated from asthma patients (DHLF) exhibit higher expression of NOX4 and α-SMA compared to fibroblasts from normal (NHLF) subjects, which were significantly increased in response to TGFβ1. Importantly, we found that *NOX4* expression is highly upregulated in experimentally induced asthma model (*D. farinae-*challenged mice) compared to controls, suggesting an essential role for NOX4 in airway remodeling. We had established earlier that TRPV4 KO mice were protected from *D. farinae*-induced lung remodeling during asthma and its concomitant increase in TGFβ lung transcripts^19^. Finally, we found that *D. farinae* challenge failed to increase expression of NOX4 and *a*-SMA as well as pathological airway remodeling in TRPV4 KO mice, suggesting that TRPV4 relays fibrotic responses via NOX4.

**Figure 7:**
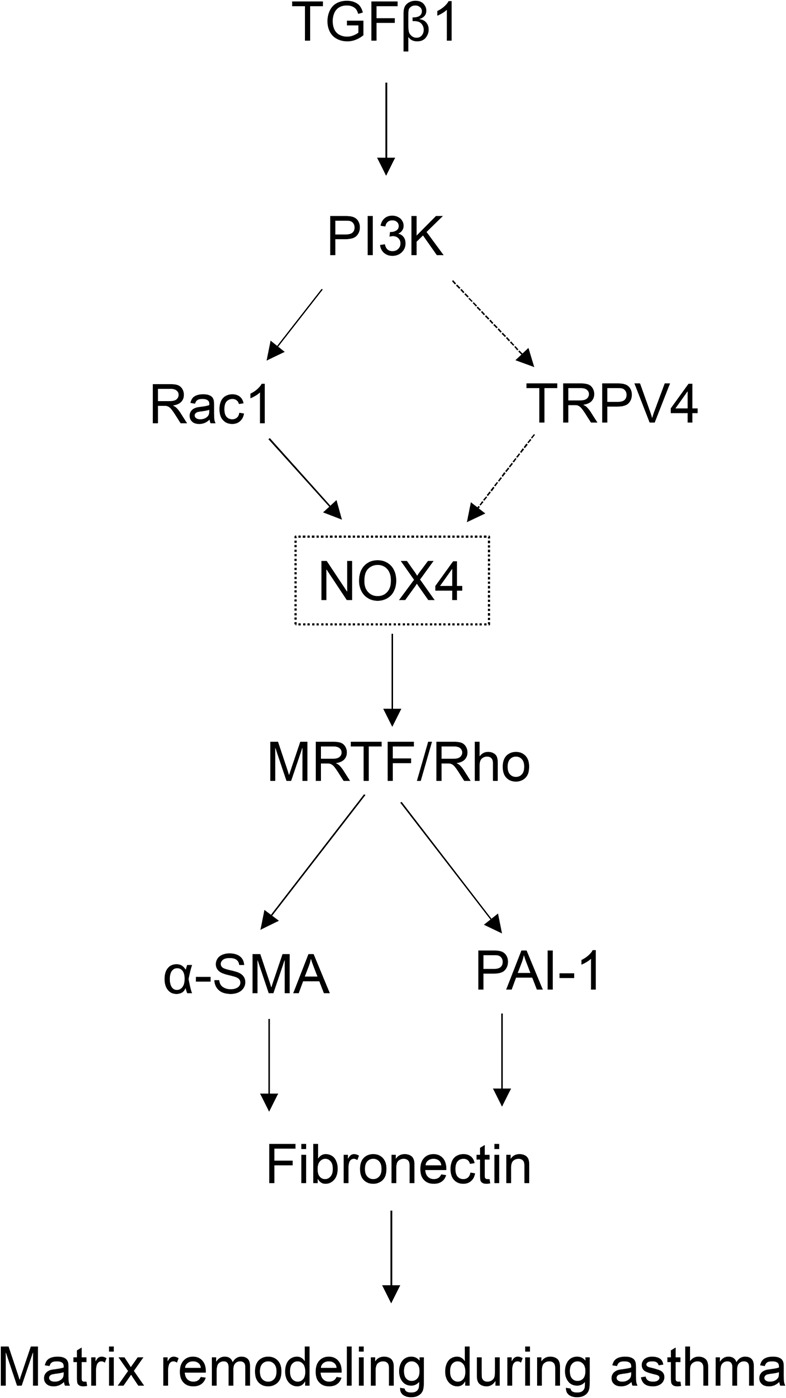
Schematic diagram depicting TRPV4 in the regulation of airway remodeling through NOX4.

In this study, we demonstrated that mechanosensitive ion channel TRPV4 integrates growth factor (TGFβ1) and redox (NOX4) signaling during lung fibroblast differentiation and airway remodeling in asthma (Fig. 7). We concluded this based on the findings that (1) TGFβ1 induced lung fibroblast differentiation through NOX4-dependent generation of ROS, (2) pharmacological or siRNA downregulation of NOX4 significantly attenuated ROS generation, MRTF-A activation, and fibroblast differentiation, (3) pharmacological inhibition or siRNA downregulation of TRPV4 significantly inhibited NOX4 expression and fibroblast differentiation, and (5) NOX4 is upregulated in fibroblasts from asthmatic subjects and airway remodeling in experimentally induced asthma, and this response is attenuated in TRPV4 KO mice. In conclusion, our results suggest that targeting TRPV4/NOX4 signaling may provide a new therapy for lung remodeling in asthma in situations where treatments focused solely on steroids or other conventional treatments have proven ineffective.

## Supplementary information

Supplementary Information.
