# Supplementary material for: Transient Receptor Potential Vanilloid channel regulates fibroblast differentiation and airway remodeling by modulating redox signals through NADPH Oxidase 4
Source: Sci Rep. 2020 Jun 17;10:9827. doi: 10.1038/s41598-020-66617-2 (PMC7299963; doi:10.1038/s41598-020-66617-2)

Transient Receptor Potential Vanilloid channel regulates fibroblast differentiation and airway remodeling by modulating redox signals through NADPH Oxidase 4

Nosayba Al-Azzam [1,3], Lakshminarayan Reddy Teegala [1], Sabita Pokhrel [1], Samrawit Ghebreigziabher [1], Tatiana Chachkovskyy [1], Sathwika Thodeti [1], Ignacio Gavilanes [1], Kayla Covington [1], Charles K Thodeti [2], Sailaja Paruchuri [1*]

[1] Department of Chemistry, University of Akron, Akron, OH, [2] Department of Integrative Medical Sciences, Northeast Ohio Medical University, Rootstown, OH, [3] Department of Physiology and Biochemistry, Jordan University of Science and Technology, Irbid, Jordan

Running Title: TRPV4 regulates fibroblast differentiation via NOX4

***Corresponding Author:**
Sailaja Paruchuri, PhD, Department of Chemistry, KNCL 406, 190 E.Buchtel Common, Akron, OH 44325; Phone: 3309722193; E-mail: sp97@uakron.edu

SUPPLEMENTAL DATA

FIGURE LEGENDS

Supplemental Figure 1. NOX4 mediates TGFβ1 -mediated α-SMA incorporation into stress fibers. NHLF were stimulated with TGFβ1 (2 ng/mL; 48 h) in the presence or absence of (A) an antioxidant NAC (10mM) or (B) NOX4 inhibitor DPI (1μM). The cells were then fixed, permeabilized and stained for α-SMA protein (red) to analyze its accumulation into stress fibers. Images of stained cells were obtained by using EVOS fluorescence microscope (X20 objective). Scale bars, 200 μm. Lower panels represent the number of α-SMA positive cells quantified by counting the number of cells with α-SMA protein expressed (red). Graphs represent means ± SEM from at least 3 independent experiments. ***P < 0.001.

Supplemental Figure 2. NAC attenuates TGFβ1 -mediated MRTF-A and PAI-1 expression. NHLF were pre-treated (30 minutes) in the presence or absence of an antioxidant, NAC (10mM), followed by treatment with TGFβ1 (2 ng/mL; 48 h). *A)* SDS-PAGE immunoblotting was performed on cell lysates using Abs specific for MRTF-A protein. Thereafter, the blots were stripped and re-probed for GAPDH. Representative blots are from a single experiment of three performed. *B)* Represents quantitative densitometric analysis of MRTF-A using AlphaView software and expressed as a percentage of control cells. Results are means + SEM from three independent experiments. *C)* PAI-1 protein (responsible for matrix degradation) was analyzed using immunoblotting, following which the blots were stripped and re-probed for GAPDH *D)* shows quantitative densitometric analysis of PAI-1 using AlphaView software and expressed as a percentage of control cells. Results are means + SEM from three independent experiments. **P < 0.01; ***P < 0.001.

Supplemental Figure 3. NOX4 regulates TGFβ1 -mediated SM22 and FN gene expression.
NOX4 protein was knocked down in NHLF by transfecting them with siRNA against NOX4 (100 nM). NHLF transfected with nonspecific (NS) siRNA were used as control. Twenty four hours after transfection, NHLF were treated with TGFβ1 (2 ng/mL; 48 h). *A)* SM22 transcript and *B)* FN transcript was analyzed by qPCR and expressed as ΔΔct. *C)* NHLF were stimulated with TGFβ1 (2 ng/mL; 48 h) in the presence or absence of indicated concentrations of a rac inhibitor EHT, and ROS (H_2O_2 generation) was measured using Amplex Red assay. After treatment, cells were washed twice in KRPG and incubated for 30 minutes with Amplex Red reagent (50 μM) and 0.1 U/mL HRP in KRPG. Fluorescence is measured expressed as relative fluorescence units (RFU) compared to control. Results are means + SEM from three independent experiments. Results are means + SEM from three independent experiments. *P < 0.05; **P < 0.01; ***P < 0.001.

Supplemental Figure 4. TRPV4 KO mice exposed to *D. farinae* exhibit fewer number of goblet cells compared to WT mice subjected to *D. farinae*.

WT and TRPV4 KO mice were treated with saline or *D. farinae* (25 mg/animal) intranasally 3 times a week for 5 weeks. Mice were euthanized 24h after the final challenge and representative photomicrographs of Periodic-acid Schiff (PAS) stained lung sections showing goblet cells (arrows; original magnification, ×40). The experiments were repeated three times with similar results. Bars, 100 µm.

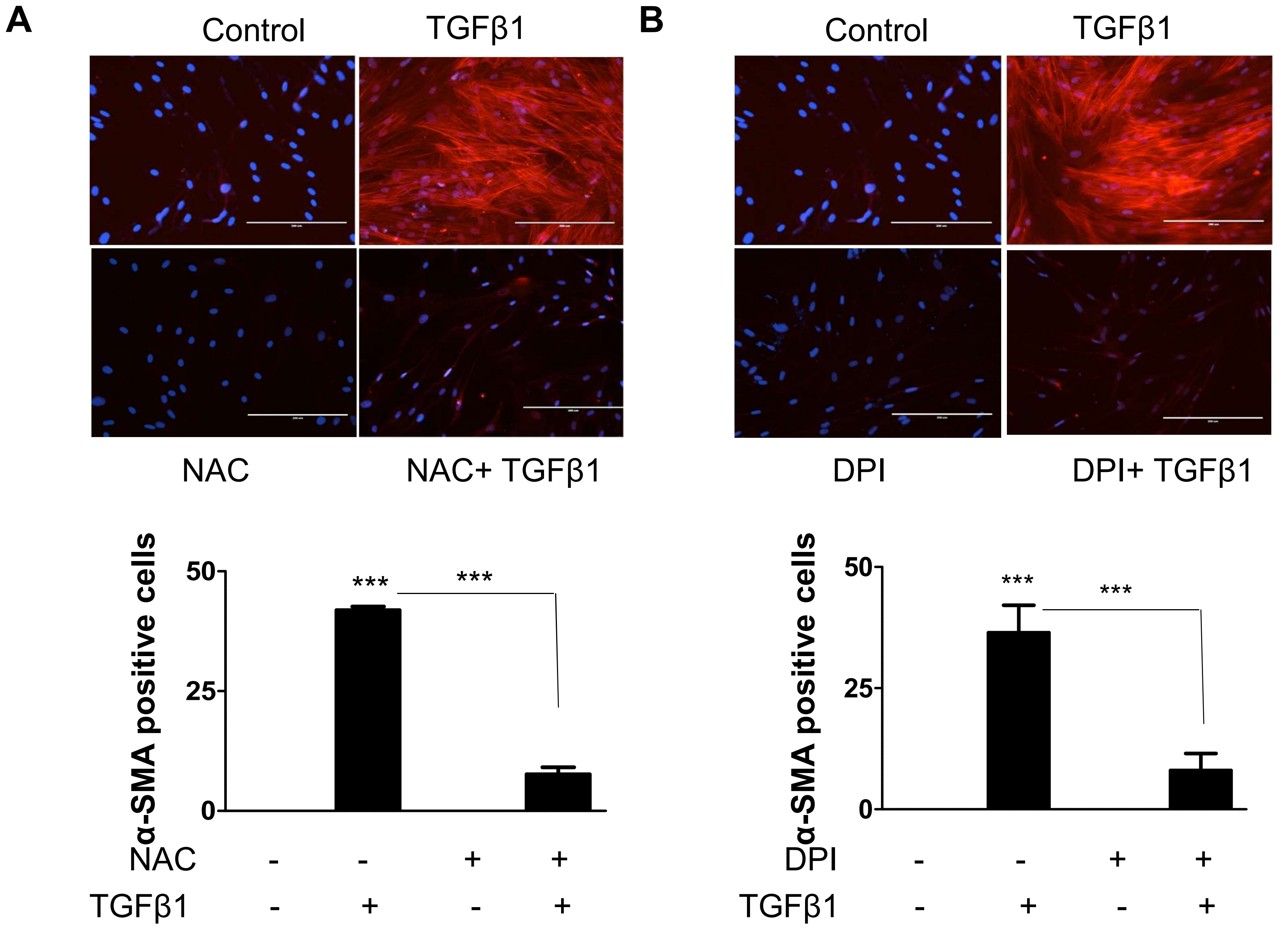

Supplemental Fig. 1

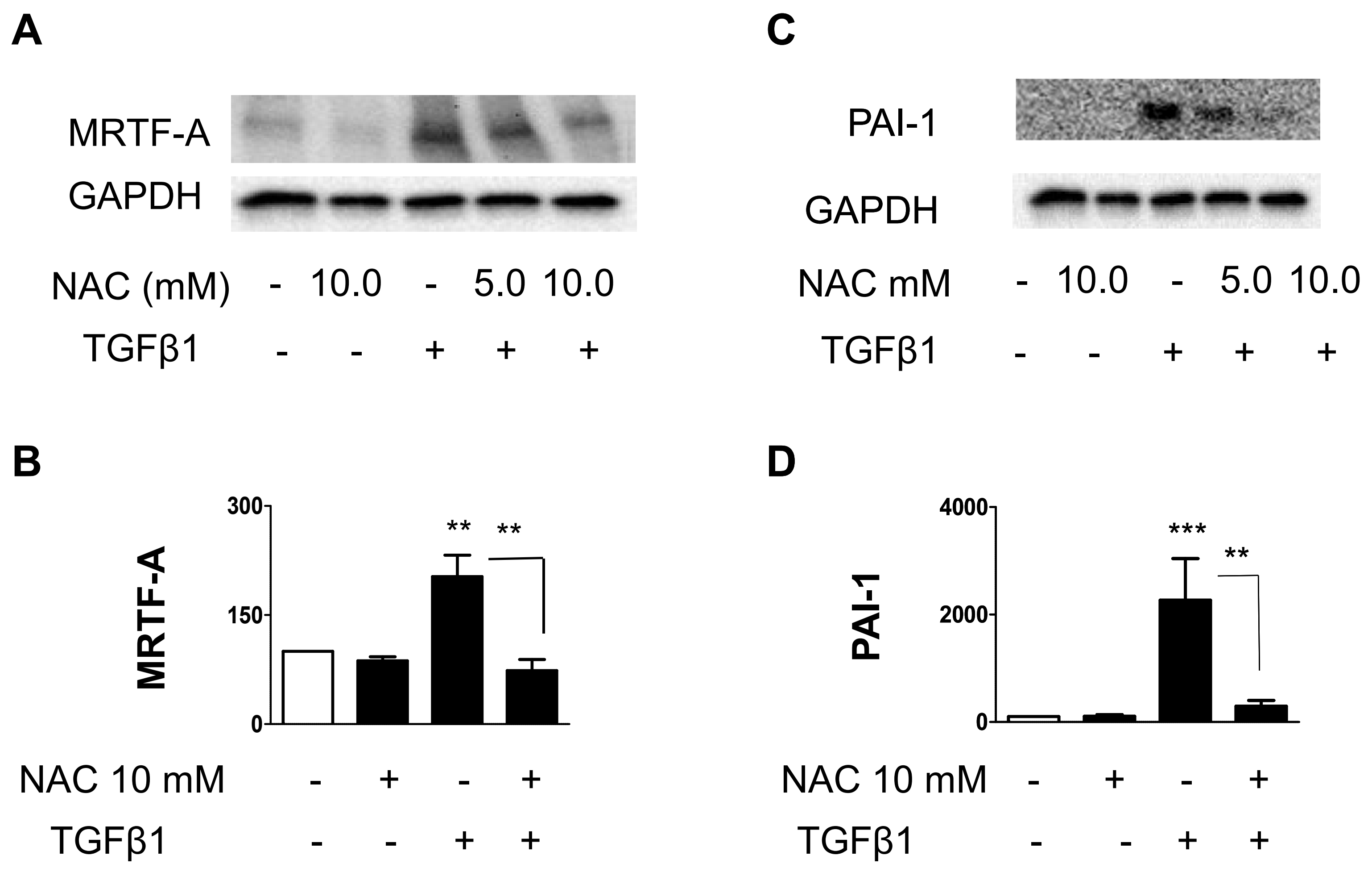

Supplemental Fig. 2

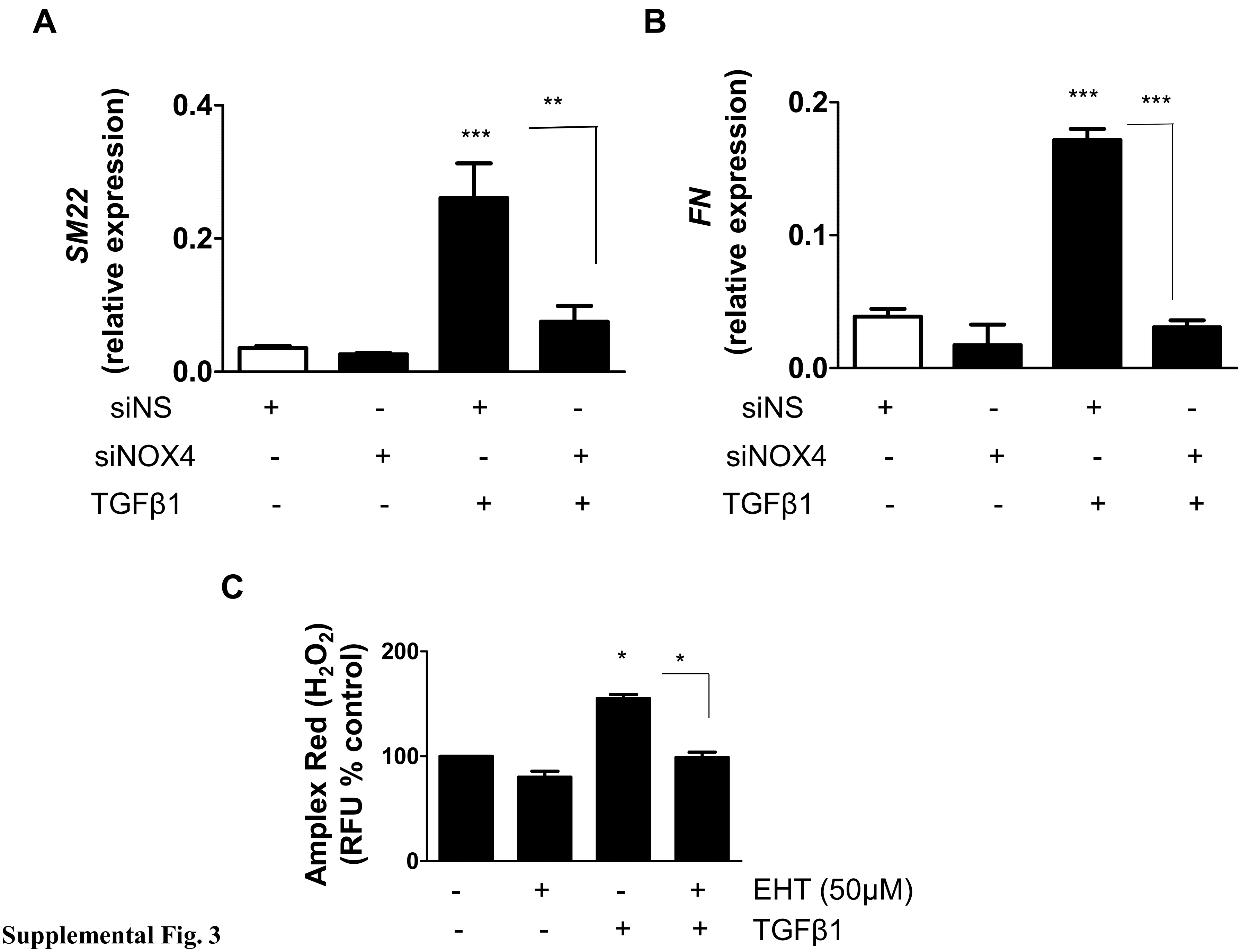

Supplemental Fig. 3

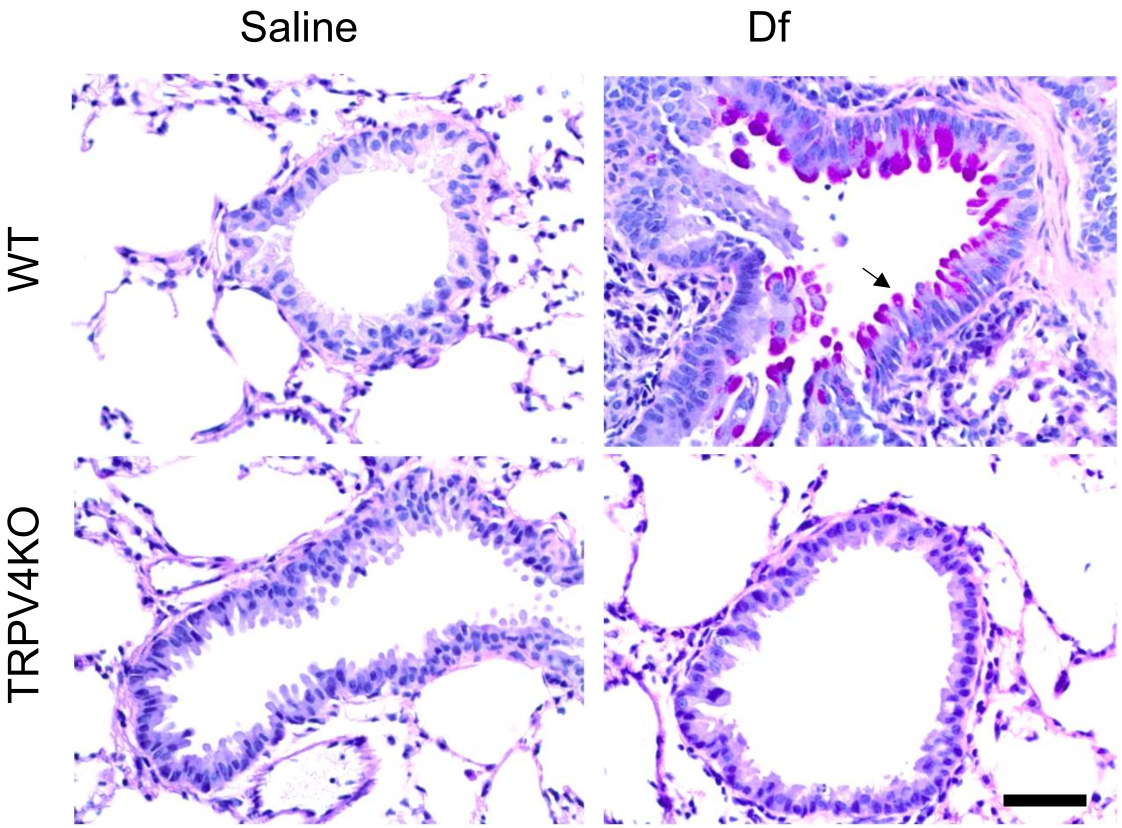

Supplemental Fig. 4

Transient Receptor Potential Vanilloid channel regulates fibroblast differentiation and airway remodeling by modulating redox signals through NADPH Oxidase 4

Nosayba Al-Azzam [1,3], Lakshminarayan Reddy Teegala [1], Sabita Pokhrel [1], Samrawit Ghebreigziabher [1], Tatiana Chachkovskyy [1], Sathwika Thodeti [1], Ignacio Gavilanes [1], Kayla Covington [1], Charles K Thodeti [2], Sailaja Paruchuri [1*]

[1] Department of Chemistry, University of Akron, Akron, OH, [2] Department of Integrative Medical Sciences, Northeast Ohio Medical University, Rootstown, OH, [3] Department of Physiology and Biochemistry, Jordan University of Science and Technology, Irbid, Jordan

Running Title: TRPV4 regulates fibroblast differentiation via NOX4

***Corresponding Author:**

Sailaja Paruchuri, PhD, Department of Chemistry, KNCL 406, 190 E.Buchtel Common, Akron, OH 44325; Phone: 3309722193; E-mail: sp97@uakron.edu

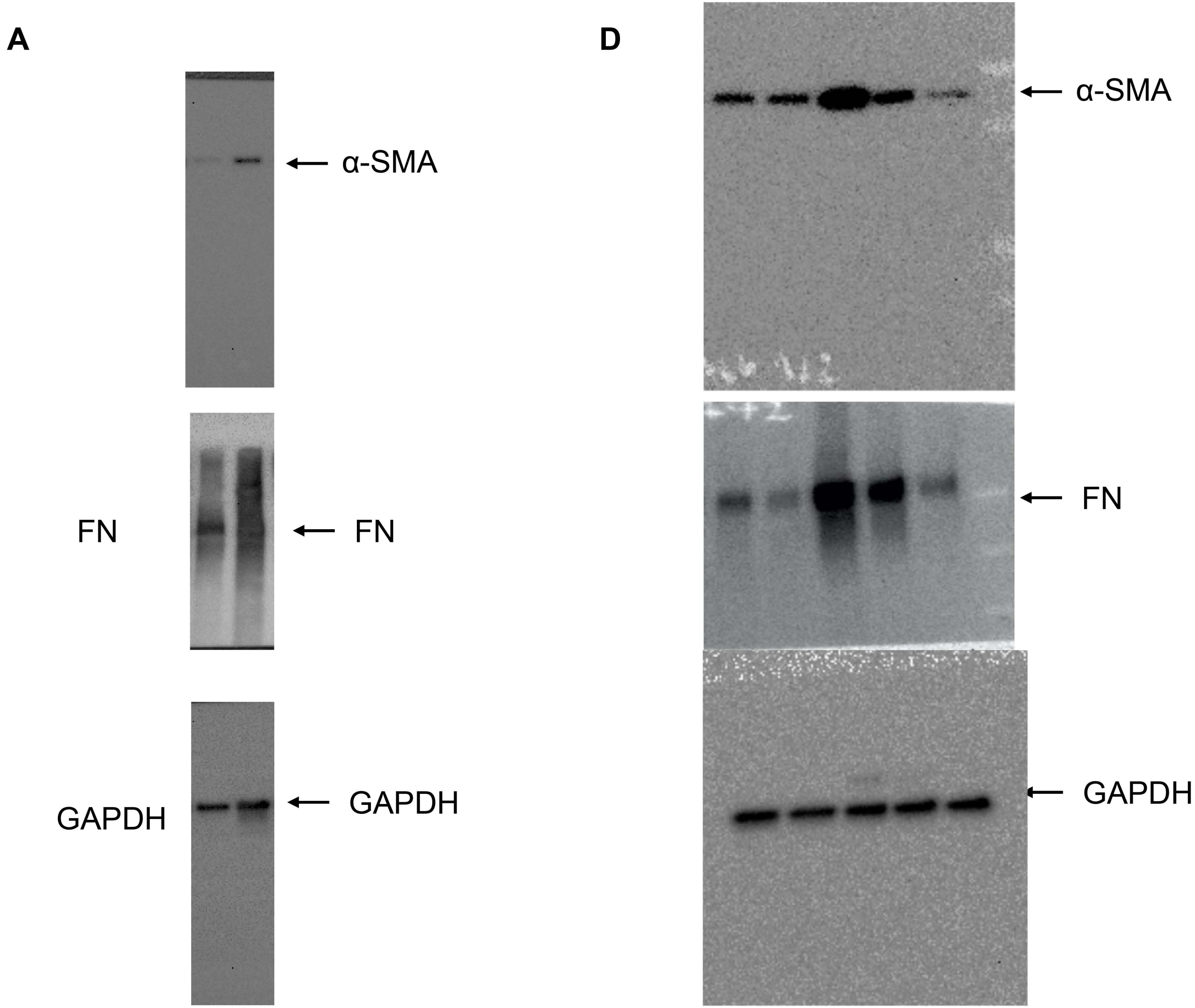

Fig. 1

Fig. 2

C

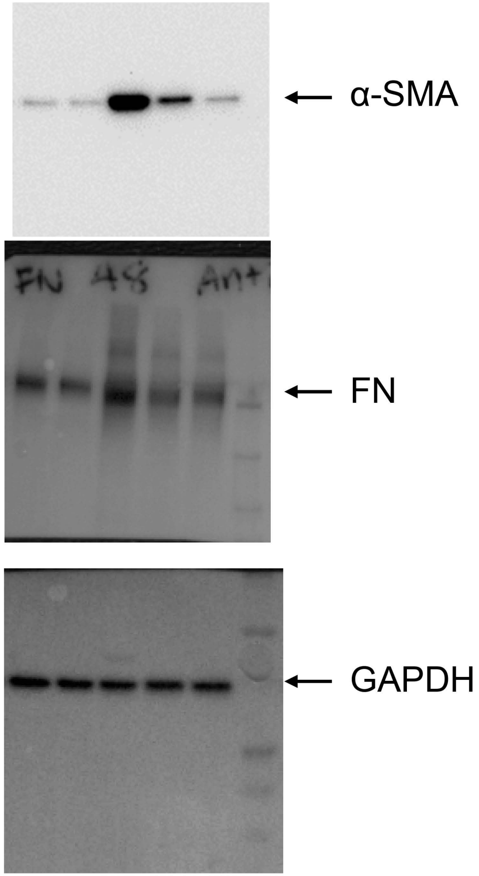

E

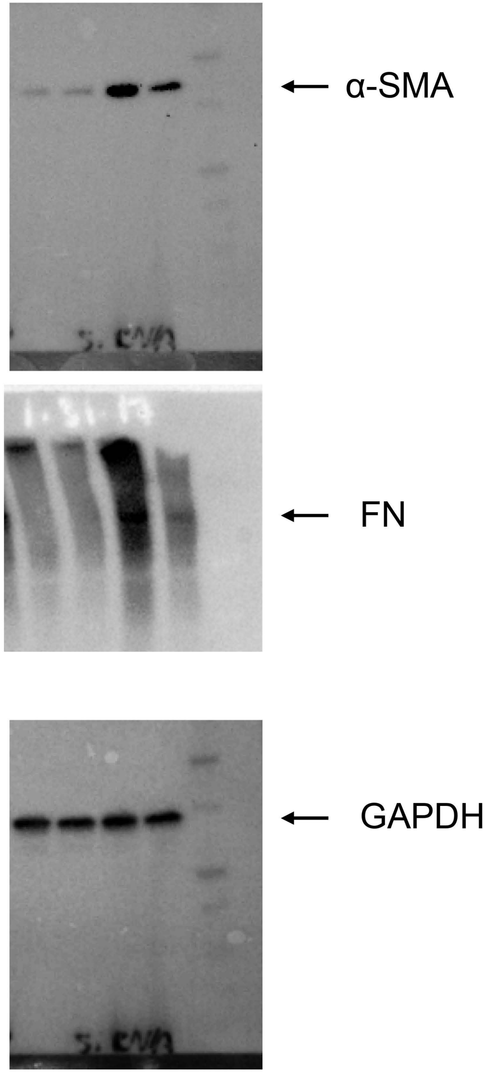

Fig. 3

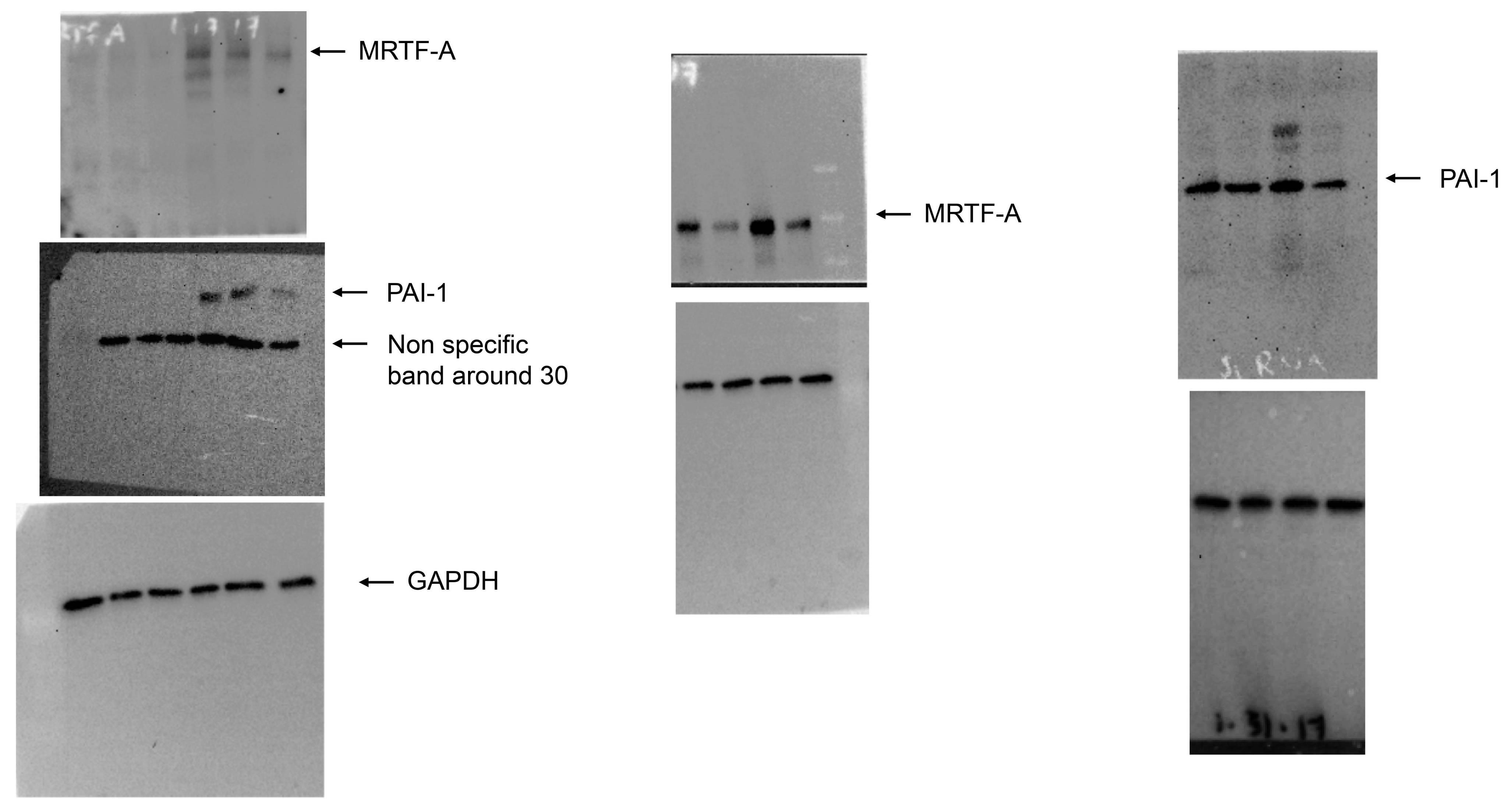

Fig. 5

D

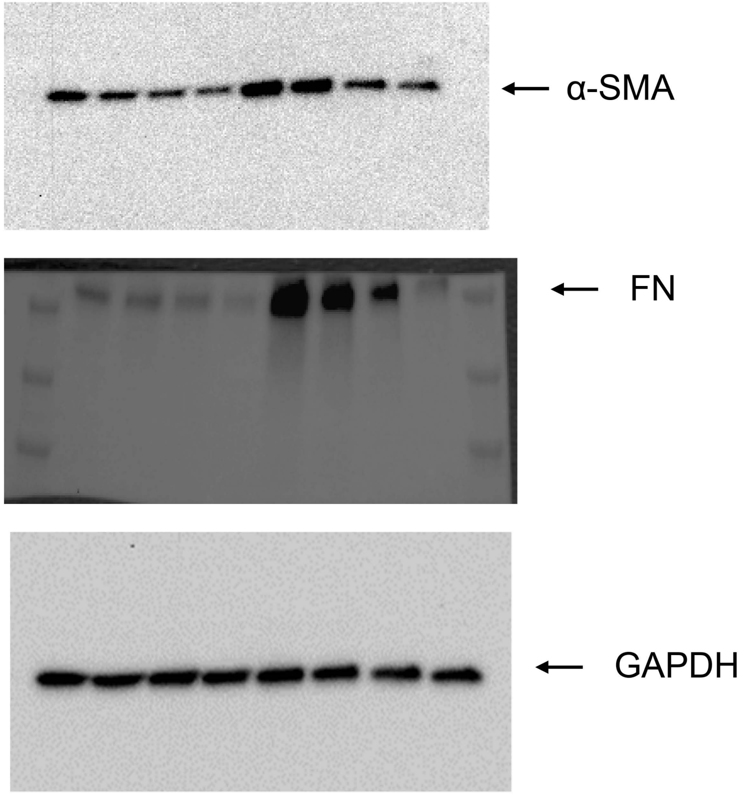

Supplement: Supplementary file 1 — Supplementary Information. [file 41598_2020_66617_MOESM1_ESM.pdf]
